# Demographics and Fracture Patterns of Patients Presenting to US Emergency Departments for Intimate Partner Violence

**DOI:** 10.5435/JAAOSGlobal-D-20-00009

**Published:** 2020-02-18

**Authors:** Randall T. Loder, Luke Momper

**Affiliations:** From the Department of Orthopaedic Surgery, Indiana University School of Medicine, Indianapolis, IN.

## Abstract

Orthopaedic surgeons are in a position to assist in identifying intimate partner violence (IPV) patients. It was the purpose of this study to analyze the demographics and fracture patterns of IPV patients in the United States.

**Methods:**

Data from the National Electronic Injury Surveillance System All Injury Program from 2005 through 2013 were used. Injuries due to domestic violence were identified, and statistical analyses accounted for the weighted, stratified nature of the data.

**Results:**

There were 1.65 million emergency department visits over nine years for IPV. The median age was 29.8 years, 83.3% were women, and 55.3% occurred at home. The major diagnoses were contusion/abrasions (43.4%), lacerations (16.9%), strain/sprains (15.6%), internal organ injuries (14.4%), and fractures (9.7%). The most common fracture involved the face (48.3%), followed by the finger (9.9%), upper trunk (9.8%), and hand (6.4%). The highest proportion of lower extremity fractures occurred in men, and upper extremity fractures increased with increasing age. The odds of fracture in an IPV patient were greatest in those sustaining an upper extremity injury (odds ratio [OR] = 6.62), lower extremity injury (OR = 6.51), upper trunk injury (4.28), and head/neck injury (OR = 3.08) compared with a lower trunk injury (referent), and women (OR = 1.80) compared with men (referent). Older patients sustaining IPV had higher odds of a fracture (the few patients 10-14 and >65 years old were excluded from this analysis).

**Conclusions:**

As this study encompasses the entire United States, these results are germane to all US orthopaedic surgeons. Knowing typical fracture patterns/locations is helpful in identifying IPV patients, although the victim may not fully divulge the history and details of the event. Identification is important for the physical and mental health of the victim, and abuse often continues if intervention does not occur. The odds of a fracture in an IPV patient are greatest when the injury involved the extremities and increased with increasing age of the patient.

Intimate partner violence (IPV) is a serious public health issue^1,2^ and of notable concern to the orthopaedic surgeon^3,4,5,6,7,8,9,10^ as well as all health care providers.^11,12^ The recognition of IPV as a substantial problem among patients with orthopaedic injuries has been acknowledged by both the American Academy of Orthopaedic Surgeons (AAOS)^13^ and the Canadian Orthopaedic Association.^14^ Orthopaedic surgeons are positioned to identify IPV patients because of their involvement in the treatment of fractures, often as the initial provider outside of the emergency department (ED). This affords the orthopaedic surgeon the ability to provide appropriate care, but also referrals for other services to prevent additional harm. Although there is an increasing body of literature on the subject of IPV to orthopaedic surgeons,^3,4,5,6,7,8,9,15^ there is little that describes the demographic characteristics, and especially fracture patterns, in IPV victims.^16,17^ It was the purpose of this study to analyze the demographics of IPV patients presenting to the EDs in the United States, especially focusing on injury and fracture patterns. Such knowledge can assist orthopaedic surgeons in identification of these patients, in addition to questioning techniques and training programs.^7,18,19,20,21,22,23^

## Methods

The data for this study come from the National Electronic Injury Surveillance System (NEISS) All Injury Program (AIP). The NEISS is a data set managed by the US Consumer Product Safety Commission (USCPSC) which collects injury data from ∼100 hospitals in the United States and its territories having an ED. It was initially designed for injuries due to consumer products. However, not all injuries are from consumer products; thus, the USCPSC selected ∼65 of these hospitals to obtain data for all injuries, regardless of the association with consumer products. This has been designated as the All Injury Program (AIP). These data are in the public domain, housed by the Interuniversity Consortium for Political and Social Research (ICPSR), and can be accessed at https://www.icpsr.umich.edu/icpsrweb/ICPSR/search/studies?q=all+injury+program. Use of this publicly available deidentified data was considered exempt by our local Institutional Review Board.

The database includes date of ED visit, sex/race/age of the injured patient, diagnosis, disposition from the ED, incident locale, body part injured, perpetrator and type of assault, and hospital size (strata). Age is also categorized into 12 different groups (≤4, 5 to 9, 10 to 14, 15 to 19, 20 to 24, 25 to 34, 35 to 44, 45 to 54, 55 to 64, 65 to 74, 75 to 84, and ≥85 years). The body part is classified into five major groups (head/neck, upper trunk, lower trunk, upper extremity, and lower extremity), as well as 26 detailed anatomic locations. The hospital strata consist of five categories, four based on size (the total number of ED visits reported by the hospital, which are small [0 to 16,830], medium [16,831 to 21,850], large [28,151 to 41,130], and very large [>41,130]) and one encompassing children's hospitals of all sizes. In 2013, there were 66 hospitals; there were 31 hospitals in the small stratum, 9 hospitals in the medium stratum, 6 hospitals in the large stratum, 15 hospitals in the very large stratum, and 5 hospitals in the children's stratum. This will thus encompass community as well as academic medical centers and trauma centers. An estimated number of patients is calculated from this weighted, stratified data set using appropriate statistical techniques.

The NEISS-AIP data for the years 2005 through 2013 were used. These years were chosen because 2013 was the last year in which data were available for collection at the time the study was done, beginning in late 2018. Data before 2005 were coded differently for many variables, making it difficult to combine the years before 2005 with those afterward. Injuries due to domestic violence were identified by the NEISS AIP codes INTENT_C = 1 (sexual assault) or 2 (other assault) and PERP = 1 (spouse/partner). Sexual assault was included as it clearly is a form of IPV and could result in a fracture. The NEISS assault and spouse/partner definitions are given in Addendum 1. Race was classified according to Eveleth and Tanner^24^ as white, black, Amerindian (Hispanic and Native American), Asian, Indo-Mediterranean (Middle Eastern and Indian subcontinent), and Polynesian. Owing to the small numbers of Polynesian and Indo-Mediterranean peoples in the data set, race/ethnicity is only reported for the white, black, Amerindian, and Asian groups. As there were very few patients in the 10 to 14 age group and those >65 years of age, they were excluded from age group analyses.

Statistical analyses were done with SUDAAN 11.0.01 software (RTI International, Research Triangle Park, North Carolina, 2013), which accounts for the weighted, stratified nature of the data. The estimated number of injuries/ED visits (N) and 95% confidence intervals of the estimate are calculated. When the actual number of patients (n) is <20 or the estimated number (N) is <1,200, such values must be interpreted with caution due to instability of the estimates. Analyses between groups of continuous data were done with the *t*-test (2 groups) or analysis of variance (3 or more groups). Differences between groups of categorical data were analyzed by the χ^2^ test. Demographic predictors of a fracture were determined with multivariate logistic regression analysis, obtaining the odds ratio (OR), and 95% confidence intervals. Incidence values were calculated using population data from the US Census Bureau the years 2005 to 2013 (http://www.census.gov/popest/archives/files/MRSF-01-US1.html., https://www.census.gov/programs-surveys/popest/technical-documentation/methodology.html). A *P* < 0.05 was considered to be statistically significant.

## Results

The number of ED visits for injuries over the 9-year period was 4,664,468, for a nationwide estimate of 275,014,511 ED visits. Injuries due to violence accounted for an estimated 19,559,460 ED visits (16,693,381 to 22,963,712) (7.1%). Of these 19,599,460 injuries, 660,155 (522,528 to 825,044) were sexual assaults and 14,313,130 (11,715,618 to 17,463,421) nonsexual assaults. Injuries due to assault from a spouse/partner (IPV) accounted for an estimated 1,654,594 (1,500,323 to 1,822,249), which represents 0.65% of all ED visits for injuries and 8.4% of injuries due to violence. The average annual incidence of ED visits for IPV per 10,000 US cohort was 6.90; 0.16 for sexual assault and 5.84 for nonsexual assault. The median age of the IPV patients was 29.8 years, 83.3% were women, 55.3% occurred at home, 58.7% sustained injuries to the head and neck, and 96.1% were treated and released from the ED. In the tables below, only the estimated N and appropriate percentages are shown. The interested reader can find the actual n and the 95% confidence limits of the estimates in Supplemental File 1, http://links.lww.com/JG9/A63.

**Figure 1:**
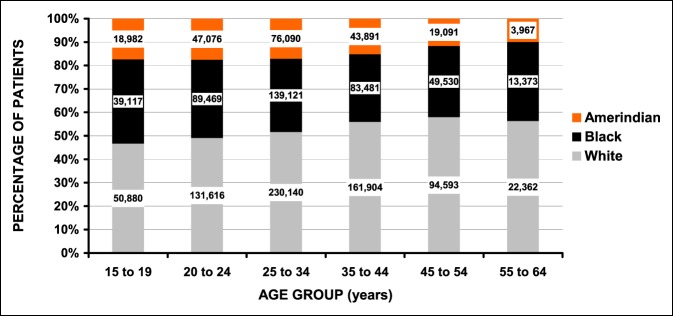
Differences by race and age group (*P* < 10^−4^) in IPV patients. The number of patients is shown in the column boxes. IPV = intimate partner violence.

Table 1 compares the sexual and nonsexual assault IPV patients. Of the 1,654,594 patients, 1,609,332 were nonsexual assaults (97.3%) and 45,262 (2.7%) sexual assaults. Those who were sexually assaulted were on the average younger (28.5 versus 32.7 years—*P* < 10^−4^) and nearly always female (99.6% versus 82.8%—*P* < 10^−4^). Although the majority of the patients were white (52.3%), the percentage of white patients was greater for the sexual assault group (66.4% versus 52.0%—*P* = 0.015). The most common anatomic area of injury for the sexual assault group was the lower trunk (81.7%) and the head/neck for the nonsexual assault group (81.7%) (*P* < 10^−4^). There were no differences in any of the other demographic variables between the sexual and nonsexual IPV groups. There were many differences by sex; however, since most IPV patients were women, detailed analyses by sex are given in Supplemental File 2, http://links.lww.com/JG9/A64. The major differences were that women (1) were younger than men (31.6 versus 33.4 years), (2) more frequently white (54.6 versus 41.1%), (3) sustained more injuries to the head/neck (60.5% versus 49.7%), and (4) less commonly admitted to the hospital (3.4 versus 6.4%). Regarding race (in addition to the differences by assault intent and sex noted above), there was an increasing percentage of white patients and concomitant decreasing percentage of black and Amerindian patients with increasing age (Figure 1). Detailed analyses by race are shown in Supplemental File 3, http://links.lww.com/JG9/A65.

**Table 1:** Demographics of Intimate Partner Violence: Sexual Versus Nonsexual Assault

Variable	All		Sexual Assault		Nonsexual Assault		
	N	%	N	%	N	%	*P*
Age (yrs)	1,654,594	—	45,262	2.7	1,609,332	97.3	—
Mean age (yrs)	32.6	—	28.5	—	32.7	—	<10^−4^
Median (interquartile)							
Age group (yrs)							
15-19	136,222	8.3	7965	18.8	128,257	8.1	0.002
20-24	331,703	20.3	9822	23.2	321,881	20.2	
25-34	551,898	33.8	12,809	30.2	539,098	33.9	
35-44	358,093	21.9	6136	14.5	351,968	22.1	
45-54	206,762	12.7	4660	11.0	202,103	12.7	
55-64	49,635	3.0	984	2.3	48,650	3.1	
Sex							
Male	276,393	16.7	191	0.4	276,202	17.2	<10^−4^
Female	1,378,202	83.3	45,072	99.6	1,333,130	82.8	
Race							
White	701,213	52.3	22,446	66.4	678,767	52.0	0.015
Black	417,729	31.2	6753	20.0	410,977	31.5	
Amerindian	211,401	15.8	4198	12.4	207,204	15.9	
Asian	9947	0.7	408	1.2	9539	0.7	
Incident locale							
Unknown	638,500	38.6	14,870	32.9	623,631	38.8	0.084
Home	914,701	55.3	27,357	60.4	887,344	55.1	
School/sports	5974	0.4	701	1.5	5273	0.3	
Street	50,524	3.1	809	1.8	49,715	3.1	
Other property	44,840	2.7	1517	3.4	43,323	2.7	
Anatomic area of injury							
Head/neck	945,075	58.7	5293	13.9	939,782	59.8	<10^−4^
Upper trunk	182,383	11.3	535	1.4	181,847	11.6	
Lower trunk	123,666	7.7	31,146	81.7	92,520	5.9	
Upper extremity	260,626	16.2	718	1.9	259,908	16.5	
Lower extremity	97,931	6.1	414	1.1	97,517	6.2	
Disposition from ED							
Rx/release	1,542,857	96.1	42,555	96.9	1,500,302	96.0	0.47
Admit	63,220	3.9	1354	3.1	61,866	4.0	
Hospital size							
Small	358,118	21.6	11,271	24.9	346,847	21.6	0.77
Medium	366,642	22.2	10,621	23.5	356,021	22.1	
Large	576,417	34.8	12,721	28.1	563,697	35.0	
Very large	350,165	21.2	9848	21.8	340,317	21.1	

ED = emergency department, N = estimated number of ED visits

**Figure 2:**
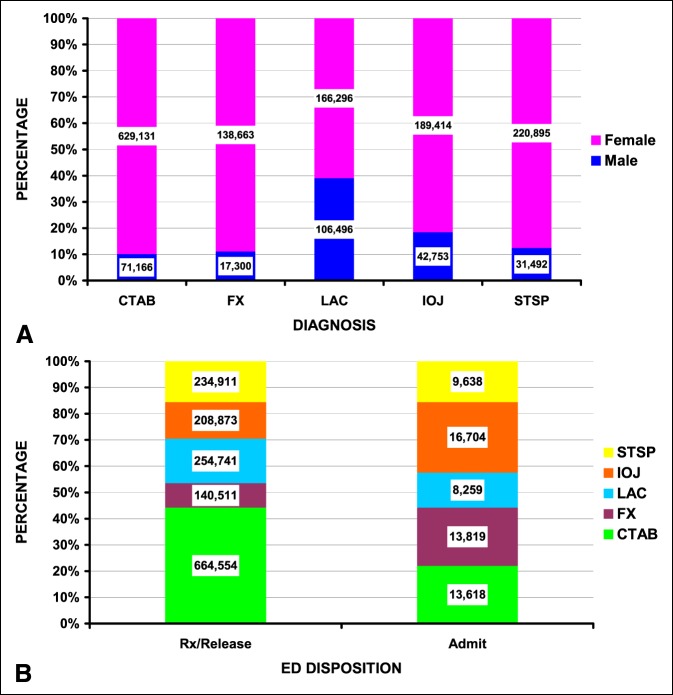
Differences by diagnosis in IPV patients. The number of patients is shown in the column boxes. CTAB = contusion/abrasion, FX = fracture, LAC = laceration, IOJ = internal organ injury, STSP = strain/sprain. **A,** By sex (*P* < 10^−4^). **B,** By hospital disposition (*P* < 10^−4^). IPV = intimate partner violence.

Table 2 shows the differences between the five major diagnoses, which accounted for 97.5% of all the injuries. These five diagnoses were contusions/abrasions (43.4%), lacerations (16.9%), strains/sprains (15.6%), internal organ injuries (14.4%), and fractures (9.7%). Lacerations were more frequent in men and blacks (Figure 2, A). Patients sustaining fractures and internal organ injuries were more commonly admitted (Figure 2, B).

**Table 2:** Demographics of Intimate Partner Violence by Injury Diagnosis

	Contusion/Abrasion		Fracture		Laceration		Internal Organ Injury		Strain Sprain		*P*
	N	%	N	%	N	%	N	%	N	%	
Age (yrs)	700,297	43.4	155,963	9.7	272,792	16.9	232,167	14.4	252,387	15.6	—
Average	32.0	—	34.0	—	33.7	—	32.7	—	31.9		<10^−4^
Median (interquartile)	29.0 (22.6-38.9)		32.0 (24.0-41.3)		31.0 (24.0-41.5)		30.4 (23.2-39.3)		28.9 (22.4-39.2)		
Age group (yrs)											
15-19	63,242	9.1	8829	5.7	18,875	7.0	16,811	7.3	25,163	10.2	
20-24	147,588	21.3	29,961	19.5	48,822	18.1	46,367	20.2	50,692	20.5	
25-34	237,704	34.3	48,438	31.5	89,953	33.3	77,720	33.8	83,030	33.6	
35-44	142,527	20.6	38,981	25.3	63,062	23.4	53,524	23.3	50,891	20.6	
45-54	82,899	12.0	23,113	15.0	38,682	14.3	28,152	12.2	30,354	12.3	
55-64	19,086	2.8	4563	3.0	10,366	3.8	7245	3.2	7056	2.9	
Sex											
Male	71,166	10.2	17,300	11.1	106,496	39.0	42,753	18.4	31,492	12.5	<10^−4^
Female	629,131	89.8	138,663	88.9	166,296	61.0	189,414	81.6	220,895	87.5	
Race											
White	306,793	53.7	73,642	57.4	89,326	39.8	95,854	52.8	116,675	57.7	<10^−4^
Black	172,349	30.2	34,171	26.6	95,706	42.7	50,411	27.8	56,427	27.9	
Amerindian	87,670	15.3	20,204	15.7	38,063	17.0	33,567	18.5	27,010	13.4	
Asian	4488	0.8	286	0.2	1113	0.5	1673	0.9	2164	1.1	
Incident locale											
Unknown	251,182	35.9	60,916	39.1	102,288	37.5	110,775	47.7	98,958	39.2	<10^−4^
Home	403,263	57.6	86,296	55.3	156,381	57.3	107,426	46.3	137,875	54.6	
School/sports	2869	0.4	317	0.2	454	0.2	612	0.3	1312	0.5	
Street	24,720	3.5	5139	3.3	5873	2.2	6232	2.7	7115	2.8	
Other property	18,217	2.6	3295	2.1	7796	2.9	7121	3.1	7119	2.8	
Anatomic area of injury											
Head/neck	383,827	56.8	81,773	52.4	167,453	61.5	201,657	86.9	74,563	32.1	<10^−4^
Upper trunk	112,074	16.6	18,086	11.6	8210	3.0	11,309	4.9	31,237	13.4	
Lower trunk	50,541	7.5	4012	2.6	5678	2.1	6542	2.8	56,322	24.2	
Upper extremity	87,944	13.0	37,855	24.3	75,960	27.9	8693	3.7	47,873	20.6	
Lower extremity	41,886	6.2	14,225	9.1	15,050	5.5	3793	1.6	22,489	9.7	
Assault type											
Sexual assault	3885	0.6	406	0.3	1407	0.5	1311	0.6	37,994	15.1	<10^−4^
Nonsexual assault	696,412	99.4	155,557	99.7	271,385	99.5	230,856	99.4	214,393	84.9	
Disposition from ED											
Rx/release	664,554	98.0	140,511	91.0	254,741	96.9	208,873	92.6	234,911	96.1	<10^−4^
Admit	13,618	2.0	13,819	9.0	8259	3.1	16,704	7.4	9638	3.9	
Hospital size											
Small	179,085	25.6	35,177	22.6	48,321	17.7	28,628	12.3	58,448	23.2	<10^−4^
Medium	176,536	25.2	32,954	21.1	54,814	20.1	38,815	16.7	53,075	21.0	
Large	210,165	30.0	54,573	35.0	98,499	36.1	113,305	48.8	86,551	34.3	
Very large	133,489	19.1	33,094	21.2	70,705	25.9	51,082	22.0	53,199	21.1	

ED, emergency department, N = estimated number of ED visits

**Figure 3:**
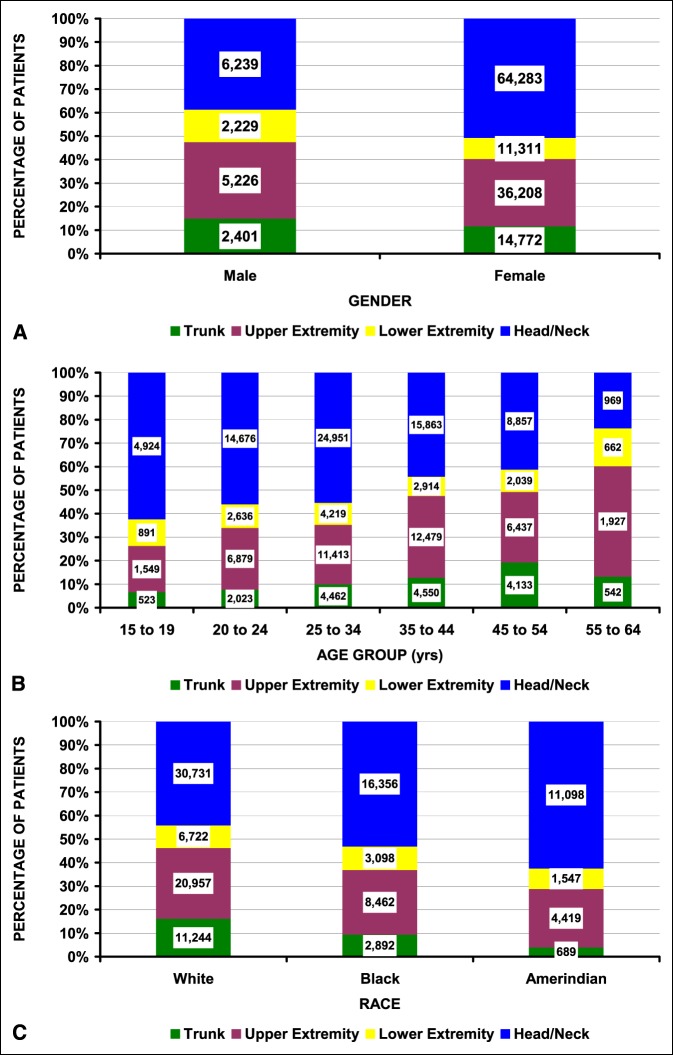
Differences in fracture location in IPV patients. The number of patients is shown in the column boxes. **A,** By sex (*P* = 0.0032). **B,** By age group (*P* < 10^−4^). **C,** By race (*P* = 0.044). IPV = intimate partner violence.

Table 3 shows detailed fracture locations. The most common fracture (involved the face [48.3%], followed by the finger [9.9%], upper trunk [9.8%], and hand [6.4%]); all other areas accounted for <5% of the fractures. The fracture locations were divided into four major groups: the head/neck, trunk, upper extremity, and lower extremity (Table 4). There were notable differences by sex, age group, race, and type of assault. Men sustained a higher proportion of lower extremity fractures compared with women (Figure 3, A). Head/neck fractures were more common in the younger age groups and decreased with increasing age; upper extremity fractures increased with increasing age (Figure 3, B). Head/neck fractures were most common in Amerindian patients with more upper extremity and trunk fractures in white patients (Figure 3, C). A fracture that occurred during a sexual assault was nearly twice as likely to be located in the lower extremity compared with the trunk.

**Table 3:** Anatomic Location of Fractures in Intimate Partner Violence Patients

Location	N	%
Skull	740	0.5
Face	68,973	48.3
Neck	809	0.6
Upper trunk	13,931	9.8
Lower trunk	3241	2.3
Shoulder	4250	3.0
Humerus	1880	1.3
Elbow	1619	1.1
Forearm	5486	3.8
Wrist	4861	3.4
Hand	9155	6.4
Finger	14,184	9.9
Femur	370	0.3
Knee	339	0.2
Tibia/fibula	2872	2.0
Ankle	3781	2.7
Foot	3523	2.5
Toe	2654	1.9

ED, emergency department, N = estimated number of ED visits

**Table 4:** Demographics of Intimate Partner Violence by Fracture Location

Variable	Trunk		Upper Extremity		Lower Extremity		Head/Neck		*P*
	N	%	N	%	N	%	N	%	
Age (yrs)	17,173	12.0	41,434	29.0	13,539	9.8	70,521	49.4	—
Average	37.3	—	35.9	—	33.9	—	32.1	—	<10^−4^
Median (interquartile)	37.5 (28.5-47.1)		35.0 (25.4-42.9)		30.8 (23.8-41.4)		29.6 (23.5-38.4)		
Age group (yrs)									
15-19	523	3.0	1549	3.7	891	6.6	4924	7.0	<10^−4^
20-24	2023	11.8	6879	16.6	2636	19.5	14,676	20.8	
25-34	4462	26.0	11,413	27.5	4219	31.2	24,951	35.4	
35-44	4550	26.5	12,479	30.1	2914	21.5	15,863	22.5	
45-54	4133	24.1	6437	15.5	2039	15.1	8857	12.6	
55-64	542	3.2	1927	4.7	662	4.9	969	1.4	
Sex									
Male	2401	14.0	5226	12.6	2229	16.5	6239	8.8	0.0032
Female	14,772	86.0	36,208	87.4	11,311	83.5	64,283	91.2	
Race									
White	11,244	65.5	20,957	50.6	6722	59.0	30,731	57.1	0.0007
Black	2892	16.8	8462	20.4	3098	27.2	16,356	30.4	0.044^a^
Amerindian	689	4.0	4419	10.7	1547	13.6	11,098	20.6	
Asian	69	0.4	49	0.1	21	0.2	123	0.2	
Incident locale									
Unknown	6161	35.9	16,970	41.0	3856	28.5	27,710	39.3	0.11
Home	9622	56.0	22,303	53.8	8474	62.6	39,201	55.6	
School/sports	0	0.0	238	0.6	0	0.0	116	0.2	
Street	800	4.7	118	0.3	865	6.4	1717	2.4	
Other property	590	3.4	743	1.8	345	2.5	1778	2.5	
Incident intent									
Sexual	158	0.9	662	1.6	231	1.7	1102	1.6	<10^−4^
Nonsexual	17,014	99.1	40,773	98.4	13,309	98.3	69,419	98.4	
Disposition from ED									
Rx/release	14,057	81.9	38,751	93.5	11,462	84.7	63,910	91.5	0.01
Admit	3091	18.0	2570	6.2	2078	15.3	5968	8.5	
Hospital size									
Small	3865	22.5	10,325	24.9	3357	24.8	13,920	19.7	0.017
Medium	4276	24.9	8440	20.4	3369	24.9	8893	12.6	
Large	5645	32.9	13,751	33.2	4371	32.3	26,446	37.5	
Very large	3369	19.6	8893	21.5	2489	18.4	15,138	21.5	

ED, emergency department, N = estimated number of ED visits

a Excluding Asian group.

Table 5 shows the results of multivariate logistic regression analyses for demographic predictors of a fracture. The parameters included in the model were simple ones that any health care provider would have available from a simple history and included age, sex, race, anatomic area of injury, and type of assault. The odds of a fracture in a patient sustaining IPV were greatest in those sustaining a nonsexual assault (OR = 4.8) compared with a sexual assault (referent); an upper extremity injury (OR = 6.62), lower extremity injury (OR = 6.51), upper trunk injury (4.28), and head/neck injury (OR = 3.08) compared with a lower trunk injury (referent); whites (OR = 1.33) compared with blacks (referent); and women (OR = 1.80) compared with men (referent). Older patients sustaining IPV had higher odds of a fracture (45 to 54 years, OR = 2.07; 55 to 64 years, OR = 1.96), (35 to 44 years, OR = 1.91; 25 to 34 years, OR = 1.59; 20 to 24 years, OR = 1.45) compared with those 15 to 19 years of age (referent).

**Table 5:** Demographic Predictors of a Fracture in Intimate Partner Violence Patients

Variable	OR	95% CI	*P*
Age group (yrs)^a^			
15-19	1.00 (R)	—	—
20-24	1.45	1.05-2.01	0.026
25-34	1.59	1.12-2.25	0.011
35-44	1.91	1.34-2.73	0.0006
45-54	2.07	1.5-2.86	<10^−4^
55-64	1.96	1.34-2.85	0.0007
Sex			
Male	1.00 (R)	—	—
Female	1.80	1.47-2.20	<10^−4^
Race			
White	1.33	1.2-1.46	<10^−4^
Black	1.00 (R)	—	—
Amerindian	1.18	0.84-1.66	0.33
Anatomic area of injury			
Head/neck	3.08	2.08-4.55	<10^−4^
Upper trunk	4.28	2.75-6.65	<10^−4^
Lower trunk	1.00 (R)	—	—
Upper extremity	6.62	4.29-10.23	<10^−4^
Lower extremity	6.51	4.26-9.95	<10^−4^
Incident type			
Sexual assault	1.00 (R)	—	—
Nonsexual assault	4.80	2.52-9.16	<10^−4^

95% CI = confidence interval of the odds ratio, OR = odds ratio, R = reference group

a Patients less than 15 and greater than 64 years of age were excluded due to small numbers.

**Figure 4:**
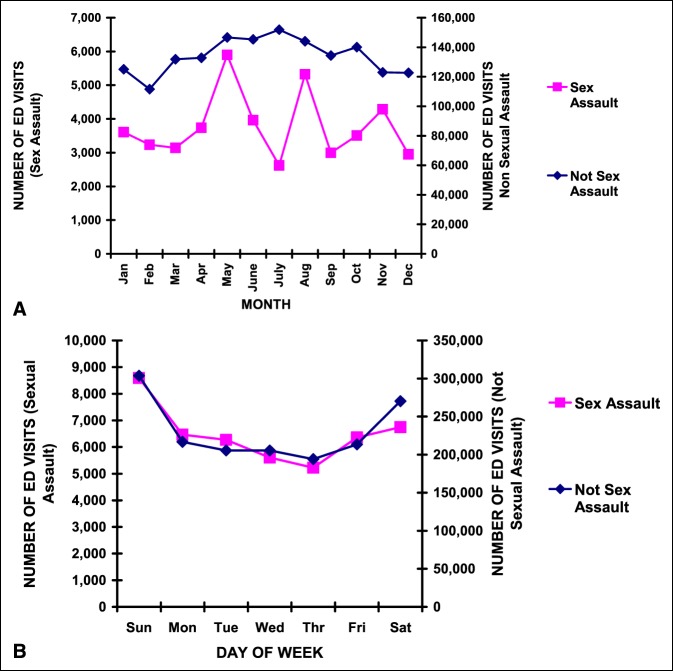
Temporal variation in IPV ED visits. **A,** By month. The differences between the sexual and nonsexual assault IPV patients were highly significant (*P* < 10^−4^). **B,** By weekday. There were no notable differences between the sexual and nonsexual assault IPV patients. ED = emergency department, IPV = intimate partner violence.

Finally, we studied temporal variation. There was a notable difference in the month of ED visit between the sexual and nonsexual assault IPV patients (Figure 4, A). The nonsexual assault group demonstrated a mild increase in the summer months. The sexual assault group demonstrated marked peaks in May and August. For both groups, more of the ED visits occurred on Saturday and Sunday (Figure 4, B).

## Discussion

There are few studies with which to compare our study. Muelleman et al,^25^ in a study from Nebraska and Kansas of 280 battered women, identified 7 fractures, all involving the face. Bhandari et al^16^ studied 263 women from Minnesota who sustained IPV. They included not only sexual and nonsexual assault, but also emotional, psychological, and financial abuse. Of these 263, 63% were white, greater than the 52.3% in this study. However, their study did not cover the entire United States, but only Minnesota. There were 144 occurrences of physical injury in 281 physically abused women; the anatomic location of the injury involved the head/neck in 40%, less than the 58.7% in this study. There were a total of 39 fractures, or 27% of the injuries, which is much higher than the 9.7% in this study (Table 2). This could be due to the fact that the patients in their study had already been referred to a nonprofit organization (Domestic Abuse Project). We surmise that the simpler strains/sprains and contusions/abrasions might not have been deemed appropriate for referral. The location of the 39 fractures in their study was the head/neck in 17 (44%), upper extremity in 13 (33%), lower extremity in 1 (3%), and trunk in 8 (20%). This is different than the results in this study, where the head/neck comprised 49.4% of the fractures, upper extremity 29.0%, lower extremity 9.8%, and trunk 12.0%. Spedding et al^26^ in the United Kingdom studied 103 female assaults due to domestic violence; of these 103, fractures occurred in 18%, more than the 9.7% in this study. The fractures in their study were located in the head/neck in 4 (21%), upper extremity in 9 (47%), and trunk in 4 (21%). Interestingly, five of the 103 women initially stated they had fallen down the stairs, but later volunteered that domestic violence was the cause of the injury. Thus, knowing typical fracture patterns/locations is helpful in identifying IPV patients, although the victim may not fully divulge the history and details of the event.

Identification of IPV is important for many reasons. The first is the physical and mental health of the victim,^12^ as well as the mental health of the abuser and both the physical and mental health of children in the relationship. Abuse often continues if intervention does not occur,^2,27^ and such violence can potentially result in homicide and/or suicide, not only to the victim but also children of the IPV victim,^28^ as well as cruelty to animals.^29^ Financial costs to society are also increased, as IPV patients consume more health care resources than those without IPV.^12,30,31^ One in 50 women presenting to an orthopaedic fracture clinic is a victim of IPV,^10^ with 64% sustaining fractures. Owing to this high prevalence of IPV-related injuries seen in orthopaedic clinics, programs are now becoming established to guide practitioners in appropriate questioning techniques regarding the potential of IPV.^3,20,22,32,33,34,35,36^ Orthopaedic surgeons are thus in a unique position to identify IPV victims. However, it is well known that an IPV victim may not always disclose the abuse/violence.^18^ Understanding the demographics and fracture patterns of IPV victims is additional knowledge that orthopaedic providers can use when discussing the possible issue of IPV with orthopaedic patients. Resources that are available to orthopaedic surgeons if an IPV victim is identified are given in a recent review.^3^ A telephone hotline, for both the United States and Canada, is 1-800-799-SAFE, which can be given to a patient and they will immediately assist the patient.

This study found that the odds of a fracture in an IPV patient are greatest when the injury involved the extremities (OR = 6.62 for the upper extremity and 6.51 for the lower extremity), followed by the upper trunk (OR = 4.28) and head/neck (OR = 3.08), compared with the lower trunk (reference). The odds of fracture increased with increasing age of the IPV victim, were greater in women (OR = 1.8) compared with men (reference), and were greatest in white, compared with Amerindian or black IPV patients. It has been previously noted that injuries to the head/neck are the most common in IPV patients.^17,25,37^ We noted the same in this study, where 58.7% of all IPV injuries involved the head/neck. Similarly, 48.3% of the fractures occurred in the face. Although the absolute number of fractures involved the face, the odds of a fracture are twice greater if the IPV victim sustained an injury to the extremities compared with the face. This new information is especially important to the orthopaedic surgeon when evaluating a fracture patient where the history seems suspect and could guide the orthopaedic surgeon to further explore/discuss the injury circumstances with the patient.

Most IPV patients in this study were women; however, men can also be victims of IPV. In this study, 16.7% of the IPV patients were men (0.4% of the sexual assaults and 17.2% of the nonsexual assaults). Men were more likely to sustain lacerations compared with other injuries (Table 2 and Figure 2, B). This 16.7% is very similar to the 17% male IPV victims in a study of 29 patients from Finland.^38^ However, our prevalence of fracture was less in men than women. When men did sustain fractures, they more commonly occurred in the extremities compared with women (Figure 3, A).

The major strength of this study is that it encompasses the entire United States, all ages, racial groups, and both sexes for patients visiting the ED for IPV injury care. As such, it reflects the entire US cohort, and these results are germane to all orthopaedic surgeons whether practicing in an urban or rural location or an academic or private situation. Many of the previously published studies regarding IPV originate from academic institutions and may not be applicable to the entire US cohort or the private practice orthopaedic surgeon.

Limitations of this study must be acknowledged. Large data sets inherently possess inaccuracy. However, the NEISS data collection protocols have 89% to 98% accuracy.^39,40^ Second, the NEISS only captures those who sought care in the ED; thus, those seeking care in physician's offices, urgent care centers, or women's health centers are not captured. This of course decreases the numbers of patients and might skew the results regarding the demographics and types of injuries. However, a patient sustaining a fracture is intuitively more likely to seek immediate care in an ED, although this is simple conjecture. Third, the event of a patient having both a fracture and a more serious injury, such as an internal organ injury (concussion, etc), cannot be ascertained as NEISS only codes one diagnosis. NEISS coders are instructed to code the diagnosis by the most severe injury, which would likely be an internal organ injury. Another limitation is the sex of the assaulting person. Although most of the cases are likely heterosexual, the percentage of nonheterosexual (LGBTQ) assaults cannot be determined from the NEISS data. Finally, another limitation is not having socioeconomic status or other social class variables. However, IPV is known to cross all socioeconomic strata.^3^

## Conclusions

As this study encompasses the entire United States, these results are applicable to all orthopaedic surgeons caring for fractures regardless of geographic location or practice type. Knowing typical fracture patterns/locations is helpful to identify IPV patients, especially when the victim may not fully divulge the history and details of the event. The odds of a fracture in an IPV patient are greatest when the injury involved the extremities and increased with increasing age of the patient.

## Addendum 1. NEISS Definition of Perpetrator and Assault/Sexual Assault

### NEISS definitions of relationshipof perpetrator to the victim as spouse or partner (includes same-sex partners)

- current partner;
- former spouse;
- former partner;
- boyfriend; former boyfriend;
- girlfriend; former girlfriend;
- father of her child; mother of his child;
- dating partner including first date (heterosexual or same sex).

### Assault, confirmed, or suspected:

Injury from an act of violence where physical force by one or more persons is used with the intent of causing harm, injury, or death to another person or an intentional poisoning by another person. This category includes perpetrators as well as intended and unintended victims of violent acts (e.g., innocent bystanders). This category excludes unintentional shooting victims (other than those occurring during an act of violence), unintentional drug overdoses, and children or teenagers “horsing” around.

### Assault—sexual: An assault as defined above that also involves

- the use of physical force to compel another person to engage in a sexual act against his or her will, whether the act is completed or not, attempted or completed sex act involving a person unable to
  1) understand the nature of the act,
  2) decline participation, or
  3) communicate unwillingness to participate for whatever reason.
- abusive sexual contact: intentional touching, either directly or through the clothing, of the genitalia, anus, groin, breast, inner thigh, or buttocks of any person against his or her will or of a person who is unable to consent (e.g., because of age, illness, disability, or the influence of alcohol or other drugs) or refuse (e.g., due to the use of guns or other nonbodily weapons, or due to physical violence, threats of physical violence, real or perceived coercion, intimidation or pressure, or misuse of authority).

This category includes rape, completed or attempted; sodomy, completed or attempted; and other sexual assaults with bodily force, completed or attempted.
